# Cancer survival in England and Wales at the end of the 20th century

**DOI:** 10.1038/sj.bjc.6604571

**Published:** 2008-09-23

**Authors:** B Rachet, L M Woods, E Mitry, M Riga, N Cooper, M J Quinn, J Steward, H Brenner, J Estève, R Sullivan, M P Coleman

**Affiliations:** 1 Cancer Research UK Cancer Survival Group, Non-Communicable Disease Epidemiology Unit, Department of Epidemiology and Population Health, London School of Hygiene and Tropical Medicine, Keppel Street, London WC1E 7HT, UK; 2 Département d'Hépatogastroentérologie et Oncologie Digestive, Centre Hospitalo-Universitaire Ambroise-Paré, 9 avenue Charles de Gaulle, F-92100 Boulogne, France; 3 Social and Health Analysis and Reporting Division, Office for National Statistics (Room FG/114), 1 Myddelton Street, London EC1R 1UW, UK; 4 Wales Cancer Intelligence and Surveillance Unit, 13th Floor, Brunel House, 2 Fitzalan Road, Cardiff CF24 0HA, Wales; 5 Division of Clinical Epidemiology and Ageing Research, German Cancer Research Center, Bergheimer Strasse 20, D-69115 Heidelberg, Germany; 6 Biostatistics Division, Hospices Civils de Lyon, Université Claude Bernard, 162 avenue Lacassagne, F-69424 Lyon Cédex 3, France; 7 Department of Social Policy, London School of Economics and Political Science, Houghton Street, London WC2A 2AE, UK

**Keywords:** survival, socioeconomic status, trends, England, Wales

## Abstract

Survival has risen steadily since the 1970s for most cancers in adults in England and Wales, but persistent inequalities exist between those living in affluent and deprived areas. These differences are not seen for children. For many of the common adult cancers, these inequalities in survival (the ‘deprivation gap’) became more marked in the 1990s. This volume presents extended analyses of survival for adults diagnosed during the 14 years 1986–1999 and followed up to 2001, including trends in overall survival in England and Wales and trends in the deprivation gap in survival. The analyses include individual tumour data for 2.2 million cancer patients. This article outlines the structure of the supplement – an article for each of the 20 most common cancers in adults, followed by an expert commentary from one of the leading UK clinicians specialising in malignancies of that organ or system. The available data, quality control and methods of analysis are described here, rather than repeated in each of the 20 articles. We open the discussion between clinicians and epidemiologists on how to interpret the observed trends and inequalities in cancer survival, and we highlight some of the most important contrasts in these very different points of view. Survival improved substantially for adult cancer patients in England and Wales up to the end of the 20th century. Although socioeconomic inequalities in survival are remarkably persistent, the overall patterns suggest that these inequalities are largely avoidable.

The survival of cancer patients in England and Wales increased for many of the common cancers during the 1970s and 1980s, but there were persistent socioeconomic inequalities in survival for most cancers in adults, even after correction for the higher background risk of death in more deprived groups (Coleman *et al*, 1999). For most cancers, survival was lower for adults resident in more deprived areas than for residents in more affluent areas – the ‘deprivation gap’ in survival. No such deprivation gap in survival was observed for cancers in children.

The main aims of this study were to extend the cancer survival trends for England and Wales to the end of the 20th century by including adults diagnosed during the period 1986–1999 and followed up to 2001, and to evaluate any changes in the socioeconomic inequalities in survival. A summary has been published (Coleman *et al*, 2004): briefly, it showed that survival continued to increase for most of the 20 common types of cancer in adults, but – again for most cancers – socioeconomic inequalities in survival either remained unchanged, or actually widened.

In this supplement, we present more extensive results on the trends and socioeconomic inequalities in survival, with a separate article on each of the 20 most common malignancies in adults.

Expert clinical commentary is provided on each set of results by leading UK clinicians who specialise in the management of each malignancy or group of malignancies. The aim was to set out in detail the possible reasons (stage at presentation, impact of various treatment modalities) for the observed changes in survival and the deprivation gap. This unique pairing of epidemiology and expert clinical analysis should provide a more complete picture of the state of cancer survival and cancer care in England and Wales.

## Structure of this supplement

Data, methods and presentation of results are discussed here, rather than in each of the 20 articles. We summarise cancer registration in England and Wales, explain how the cancers were defined, how a deprivation category was assigned to each patient and how the life tables were constructed. We describe the range of analytic approaches and the standard tables and graphics for the results.

### Survival articles

Each article contains a brief overview of the epidemiology (frequency, age–sex incidence, causes, treatment and mortality) of the cancer, including major shifts in diagnosis or treatment during the 1990s. Major socioeconomic differences in trends in incidence or mortality in England and Wales are noted. Changes in the clinical or pathological definition of disease or in the intensity of case finding are relevant for some cancers (e.g., prostate, bladder and myeloma).

We present overall national trends in cancer patient survival up to 10 years after diagnosis, adjusted for deprivation, for the periods 1986–1990, 1991–1995 and 1996–1999. Short-term predictions of survival for patients diagnosed during 2000–2001 are presented. Socioeconomic inequalities in survival in the same three calendar periods are also presented, along with short-term predictions of the deprivation gap in survival up to 10 years after diagnosis for patients diagnosed during 2000–2001.

We offer a brief interpretation of these results from the perspective of epidemiology and public health.

### Clinical commentaries

Each article on population-based survival patterns for malignancies of an organ or organ system is followed by a clinical commentary. These two views of cancer patient survival are not often presented together.

The commentaries cover the clinical presentation, diagnosis, investigation and treatment of each cancer, and any changes since the late 1980s, especially any progress in diagnostic investigation or new treatment strategies that may have had an impact on prognosis. The impact of the introduction of mass screening for cancers of the cervix and breast in the late 1980s is discussed (mass screening for bowel cancer did not start until 2006). The articles also cover the introduction and dissemination of clinical guidelines for diagnosis and management.

The commentaries discuss the extent to which the trends and socioeconomic inequalities in cancer survival could be explained by socioeconomic differences in either individual factors or healthcare system factors. These factors include the speed with which patients seek healthcare, the efficiency of referral for investigation, stage at diagnosis, access to specialist treatment or care, compliance with treatment, comorbidity and nutritional or immune status. Some commentaries discuss how insights from the clinician's practice in the treatment of individual patients can be translated to the population level, and whether recent changes in clinical practice may be expected to produce significant improvements in survival at a population level over the next 5 to 10 years.

## Material and methods

This section provides a summary of cancer registration in England and Wales, an explanation of how the cancers and deprivation groups were defined, and a description of how the data were prepared for analysis.

The cancer data comprise anonymised individual records for all cancer patients diagnosed during 1986–1999 and registered by one of the population-based cancer registries covering the whole of England and Wales.

### The cancer registration system

Cancer registries collect a small standard data set for all cancer patients in a defined population. A regional cancer registration system has covered the entire population of England and Wales since 1962, and the National Cancer Registry has collated the regional data to produce national information on cancer patterns since then. The National Cancer Registry has been linked to the National Health Service Central Register (NHSCR) since 1971. NHSCR provides notification of the eventual death of all cancer patients registered since 1 January 1971 whose record was successfully ‘flagged’ at NHSCR with details of the initial cancer registration. The National Cancer Registry also receives information about the death of all people for whom cancer was mentioned on the death certificate. In 1999, nine regional cancer registries were operating in England, alongside the national cancer registry for Wales. Data collection methods vary between cancer registries, but the principal sources are hospital in-patient records and pathology records.

### Definition of cancer

Cancers were variously defined by their anatomic location (site), their microscopic appearance (morphology) and whether their behaviour was classified as benign, *in situ* or malignant. Only malignant tumours were included. Solid tumours were generally grouped for analysis on the basis of their anatomic location. During 1986–1999, two coding systems for the site of tumours were used by the National Cancer Registry: the ninth revision of the International Classification of Diseases (ICD-9) for 1986–1994, and ICD-10 for cases diagnosed in 1995 or later (World Health Organisation, 1977, 1994). Coherent groups were constructed by bridging the classifications.

### Data extraction

Before extraction of the data for survival analysis, special efforts were made to ensure that the National Cancer Registry was as up-to-date as possible. Data sets that had been recently submitted by regional registries were checked for late registrations of cancer patients diagnosed up to 31 December 1999. The National Health Service Central Register was checked to ensure that any deaths occurring in cancer patients eligible for inclusion in the analysis had been linked to the corresponding cancer registration. The data were extracted from the National Cancer Registry on 5 November 2002, when the vital status (alive, emigrated, dead and not traced) at 31 December 2001, the closing date for survival analyses, was known for 98.4% of patients.

### Data quality

Standard criteria were used to determine the eligibility of tumour records for analysis. Records that did not meet basic criteria of data quality, residence, tumour morphology and behaviour were excluded. Thus of the 3 million registrations of a neoplasm in adults in England and Wales during 1986–1999, some 11% were of an *in situ* neoplasm, mainly of the cervix (Table 1). Exclusion of these and other registrations considered ineligible left the records for some 2.6 million adults (aged 15 years or more) registered with one of the twenty types of malignant, invasive, primary neoplasm for analysis.

Eligible tumour records were subjected to further exclusion criteria, including age (15–99 years at diagnosis), unknown vital status or sex, duplicate registration, multiple primary malignancy, synchronous tumours, invalid dates or sequences of dates and patients whose survival was zero or unknown. Ten per cent of patients were excluded because their recorded survival time was zero; these were mainly patients whose tumour registrations were made from a death certificate only (DCO), and for whom the duration of survival was therefore unknown. Other patients were excluded because it was not their first primary cancer (3.2%) or for other reasons such as unknown vital status (1.6%; Table 1). The analyses included 2.2 million adults (84.1% of those eligible) who were diagnosed with a first, primary, invasive malignancy during 1986–1999 and followed up to 31 December 2001.

### Mortality and survival

The observed probability of death among cancer patients has two components: the probability of death from the cancer of interest (the excess mortality) and the probability of death from all other causes (the background mortality).

The probability of survival, the complement of mortality, can be resolved into the same two components. The parameter of interest is the survival from cancer in the absence of other causes of death, or the ‘net probability of survival’ (Estève *et al*, 1994). Net survival can be estimated in two ways. In cause-specific survival, only deaths certified as due to the cancer of interest are considered as events in the survival analysis, and deaths from other causes are censored, along with persons lost to follow-up. This approach is not suitable for population-based data, however, because information on the underlying cause of death, especially at long intervals of time after diagnosis, is not sufficiently reliable to enable robust comparisons of survival over time and place. Relative survival, the alternative approach used here, is the ratio of the observed probability of survival and the probability of survival that would have been expected if the cancer patients had simply been subject to the background mortality of the general population in which they live, given the same initial distribution of prognostic factors such as age, sex, geographical area, calendar period and socioeconomic factors (Berkson and Gage, 1950; Cutler and Ederer, 1958; Ederer *et al*, 1961).

### Deprivation

The National Cancer Registry for England and Wales contains no information about the income or socioeconomic status of individual cancer patients. Instead, as with earlier analyses (Dickman *et al*, 1998; Coleman *et al*, 1999) and more recent work (Kaffashian *et al*, 2003; Shack *et al*, 2007), ecological measures of deprivation were used, on the basis of characteristics of the small area in which each patient was resident at the time of diagnosis.

The ranked national distribution of area deprivation scores was divided into five equal categories by quintiles, and patients were assigned a deprivation category from one (most affluent, or ‘rich’) to five (most deprived, or ‘poor’), on the basis of their postcode of residence at diagnosis. Two deprivation measures were used. For patients diagnosed during 1986–1995, we used the Carstairs score from the 1991 census, on the basis of car ownership, overcrowding, unemployment and social class IV or V of the head of household (Carstairs and Morris, 1989; Carstairs, 1995, 2000) in each electoral ward, with an average of 2000 households. Data were not available for all components of the Carstairs score after the 1991 census, and the geographic distribution of deprivation changed substantially during the 1990s, particularly for unemployment (Department for Transport Local Government and the Regions (DTLR) and Social Exclusion Unit, 2001). The most appropriate index of deprivation for patients diagnosed during 1996–1999 was the income domain score, a component of the Indices of Multiple Deprivation (IMD) 2000, derived separately for England (Department of the Environment Transport and the Regions, 2000) and Wales (National Assembly for Wales, 2000). The electoral ward was the smallest geographic area for which the IMD could be derived in England. In Wales, it was the electoral division, an area of similar mean population. The English and Welsh income domain scores are closely equivalent, and the impact of categorising the deprivation of cancer patients in Wales with the Welsh IMD instead of the English IMD was judged minimal by the developers of both indexes (Woods *et al*, 2005a).

**Figure 1:**
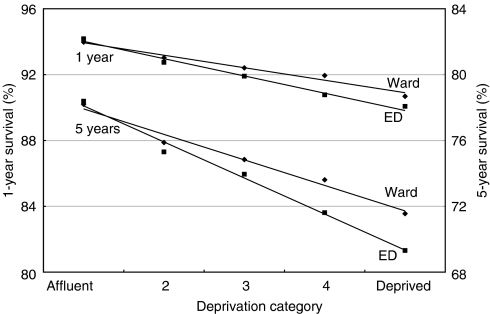
Attenuation of the deprivation gradient in 1- and 5-year survival (%) by the use of larger geographic units to assign the deprivation category: Carstairs scores on the basis of census Enumeration Districts (ED) and electoral wards, women diagnosed with breast cancer, England and Wales, during 1991–1995. (Census Enumeration Districts have populations of approximately 500 persons (200 households); electoral wards have populations that are approximately ten times larger (see text).)

In the *Cancer Survival Trends* monograph (Coleman *et al*, 1999), the deprivation gap in survival for patients diagnosed up to 1990 was estimated using the census Enumeration District (ED) as the geographic basis for assigning patients to a deprivation category. EDs contain on average only 200 households, but electoral wards (2000 households) were the smallest geographic unit for which the IMD was available in later years. Therefore, in all the analyses reported here, we used the electoral ward as the geographic unit. We evaluated the impact of this change on the deprivation gap in survival: for 29 of 34 sex-site combinations, the gap on the basis of ward Carstairs scores was smaller than on the basis of ED Carstairs scores (data not shown). The pattern for breast cancer is a typical example (Figure 1) (Woods *et al*, 2005a).

Similarly, use of the income domain score instead of the Carstairs score as the basis for determining the deprivation category, both at ward level, led to virtually identical estimates of the deprivation gap (data not shown). This implies that the population of the geographic unit is more important than the index of deprivation in measuring the deprivation gap in survival.

These changes in the index of deprivation and in the geographic basis of its assignation to cancer patients mean that estimates of the deprivation gap in survival in this supplement are not precisely comparable with those published for patients diagnosed up to 1990 (Coleman *et al*, 1999). As expected, the dilution effect of using larger geographic units in an ecological analysis tended to attenuate the survival gradient slightly. The inclusion in these analyses of patients diagnosed during 1986–1990, along with those diagnosed during 1991–1999, nevertheless enables trends in the deprivation gap in survival in the 15 years up to the end of the 20th century to be evaluated on a consistent basis.

### Abridged life tables

We constructed life tables centred on 1990–1992 and 1997–1999 for each sex and each deprivation category. For 1990–1992, the denominators were 1991 census population counts by sex and 5-year age group for each electoral ward in England and electoral division in Wales. No such small-area denominators were available for 1997–1999, and they were derived by applying Local Authority age distributions from the 2001 census for England to the populations of each ward in 1997 (Department of the Environment Transport and the Regions, 2000; Office for National Statistics, 2003). Details have been published (Coleman *et al*, 2004). For Wales, the counts of patients registered with general practitioners by single year of age and sex were available for each electoral division from the National Health Service Administrative Register.

The numbers of deaths registered in England and Wales by 5-year age group and sex were obtained from the Office for National Statistics, for each small area and each year during 1990–1992 and 1997–1999. Only 0.35% of deaths in men and 0.2% in women could not be attributed to a small area, and these were excluded.

The data on deaths and populations were aggregated across each of the five deprivation categories for England and for Wales. The annual average numbers of deaths by age, sex and deprivation category during 1990–1992 and 1997–1999 were divided by the corresponding populations for 1991 and 1998, to produce sets of mortality rates (abridged life tables) by 5-year age group, sex and deprivation, for each calendar period, both for each country and for England and Wales combined. Construction of life tables centred on a given year with the average annual number of deaths over a 3-year period is standard actuarial practice, to obtain a more robust estimate of deaths in age-sex groups with low mortality.

### Complete life tables

**Figure 2:**
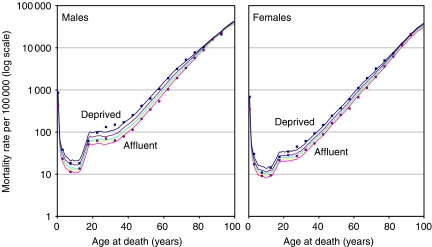
Observed and fitted mortality rates per 100 000 (log scale) by age, and by deprivation category based on the income domain of the Indices of Multiple Deprivation 1998: males and females, England and Wales, 1998. (Data points – observed mortality rates by 5-year age group (abridged life tables); only the data points for the most affluent and the most deprived categories are shown. Continuous lines – fitted mortality rates by single year of age (complete life tables); all five deprivation categories.)

The abridged life tables were smoothed, translated into complete (single-year-of-age) mortality rates and extended up to 100 years of age, using a reducible four-parameter model life table system (Ewbank *et al*, 1983), constrained to three independent parameters, which modelled the overall level of mortality (the intercept, *α*), the balance of mortality between young and old ages (the slope, *β*), and the influence of the youngest ages on the overall mortality (*κ*) (Coleman *et al*, 1999). This approach reduced the impact of minor inconsistencies in the population denominators for 1998 (Woods *et al*, 2005b) and enabled mortality estimates to be modelled for persons over 85 years of age, for whom no age-specific details were available in the abridged data. The English Life Table for 1991 by single year of age was used as the standard against which other life tables were modelled (Government Actuary's Department, 2002). A comparison of the observed (abridged) and smoothed (complete) mortality rates in 1998 for males and females in the most affluent and most deprived categories is shown in Figure 2.

National life tables by calendar period and sex were used to examine overall cancer survival patterns. National life tables by deprivation, period and sex were used to examine socioeconomic differences in survival. Expected mortality for the period 1986–1995 was derived from the 1990–1992 life tables. The 1997–1999 life tables were used for the period 1996–2001.

### Relative survival

Relative survival directly reflects the excess mortality among the cancer patients (Estève *et al*, 1990). All deaths during the study period are included, and information on the cause of death is not required. This is an advantage, especially with population-based data. Relative survival permits long-term survival to be examined, as it corrects for the increasing background mortality with ageing of the patient cohort. It is the only viable approach for the comparison of cancer survival between population groups or countries, as it also enables compensation for the wide international differences in background mortality (Micheli *et al*, 2003a).

At each time (*t*) since diagnosis, the relative survival from the cancer, *S*_*r*_(*t*), is: 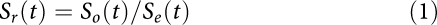 where *S*_*o*_(*t*) and *S*_*e*_(*t*) are the observed and expected probabilities of survival, respectively, each cumulated over all the successive intervals within which survival is estimated, up to time *t*.

On the mortality scale, the observed mortality rate (*λ*_*o*_) within a given interval since diagnosis is equal to the sum of the background mortality rate (*λ*_*e*_) and the excess mortality rate (*λ*_*c*_), which is considered as independent: 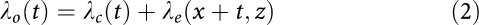 where *x* is the age of the patient at diagnosis. The background mortality rate is the expected mortality rate for a person in the general population at age (*x*+*t*), for the set (*z*) of factors such as sex, country, year of death and deprivation category for which life tables are available.

We used a relative survival model in which the excess mortality hazard is estimated with a maximum likelihood approach from individual tumour records (Estève *et al*, 1990). The baseline excess hazard is estimated as a simple step function: 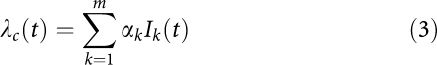 where *α*_*k*_ is the excess mortality hazard in the *k*th interval since diagnosis, and *I*_*k*_(*t*) is an indicator, which is set to 1 in the *k*th interval and 0 otherwise. This allows the excess mortality hazard to change stepwise between the *m* prespecified intervals of time since diagnosis, but to remain constant within each interval.

### Interval structure

The assumption of a constant excess hazard of death from the cancer may not be justified if time intervals are too wide, particularly in the first few months after diagnosis. The lethality of many cancers is highest then, before falling progressively, and the interval structure must reflect this. In the first year after diagnosis, therefore, we assessed survival probabilities within each consecutive month up to 6 months, then 3-monthly intervals. Estimates were made at 6-monthly intervals during 2 to 5 years, then yearly up to 10 years. It may be impossible to estimate the excess mortality if there are very few patients or very few deaths within a given time interval, because the sparsity of data prevents convergence of the maximum likelihood algorithm. In such cases we reanalysed the data after progressive regrouping of the time intervals, provided that estimates were available at exactly 1, 5 and 10 years after diagnosis.

### Design of survival analyses

We estimated relative survival up to 10 years after diagnosis for each cancer and sex, by calendar period of diagnosis and deprivation category, using one of three designs: cohort, complete and hybrid. Analyses were done with the programme *strel*, which is in the public domain (Cancer Survival Group, 2006).

**Figure 3:**
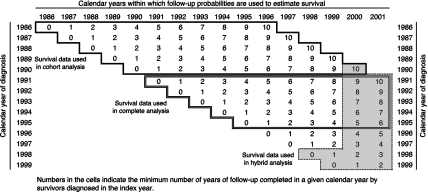
Schema to show how the follow-up data contributed by cancer patients diagnosed in each year during 1986–1999 contribute to the survival estimates for successive calendar periods using cohort, complete and hybrid approaches (see text).

Follow-up ended on 31 December 2001, so all patients diagnosed during 1986–1990 contributed to the estimate of survival up to 10 years after diagnosis (Figure 3). The probability of survival up to 10 years was estimated conventionally, as the cumulative product of the probabilities of surviving each consecutive follow-up interval from diagnosis up to 10 years. This corresponds to the classical observation of a fixed cohort until all patients have been followed up for at least the duration of survival being estimated.

For patients diagnosed during 1991–1995, the minimum duration of follow-up was 6 years, but the number of patients contributing to the estimate of survival is progressively smaller for longer periods of follow-up. Thus, only patients diagnosed during 1991–1992 can provide an estimate of the probability of survival in the tenth year after diagnosis (i.e., after at least 9 full years of follow-up). All the follow-up data for patients diagnosed 1991–1995 can still be used, however, in the ‘complete’ design (Figure 3).

For patients diagnosed during 1996–1999, the conditional probability of survival in the fifth year after diagnosis was based only on those diagnosed during 1996–1997. Ten-year survival could not be estimated in cohort fashion, because none of the patients had been followed up for 10 years. Longer-term survival for patients diagnosed more recently can be predicted with the period approach (Brenner and Gefeller, 1996), by using the most recent period for which follow-up data are available for patients who have in fact been followed up for 10 years. The principle is identical to that used for ‘estimating’ life expectancy at birth. Death rates from the most recent available year or period are used to predict (rather than to estimate) the life expectancy of a baby born in that year or period, on the assumption that she/he were subject to the most recently observed risks of death at each age up to 80 years or more, that is, up to 80 or more years into the future. No one expects last year's death rates to remain fixed for 80 or more years, so life expectancy at birth is a convenient summary of recent mortality rates and, if death rates continue to decline, it will prove to be a conservative estimate of life expectancy for last year's birth cohort, if we were to wait long enough to follow up its members to measure their life expectancy directly.

The period approach to survival analysis adopts a parallel tactic to provide short-term predictions of cancer survival. Follow-up data are used from the most recent calendar period for which such data are available up to, say, 10 years after diagnosis, to produce a cumulative probability of survival up to 10 years. The follow-up data do not relate to a fixed cohort of patients (Figure 3). A period estimate of 10-year survival is a short-term prediction of survival for patients diagnosed in that period, on the assumption that they will experience the most recently observed conditional probabilities of survival in each year up to 10 years since diagnosis. Because survival is generally improving, a period estimate tends to be a conservative estimate of the eventual 10-year survival of that cohort of patients, when in due course we have observed their follow-up for long enough to measure it directly.

A period estimate of survival from these data would have been on the basis of the follow-up observed in the years 2000–2001 for cancer patients diagnosed in the period 1990–1999, because no incidence data were available for the last 2 years. In such a situation, conventional period analysis leads to biased estimates of survival and lack of statistical power. The optimal analytic approach is a hybrid of the cohort and period approaches (Brenner and Rachet, 2004), in which the most recent follow-up data for up to 10 years are combined with follow-up data for the first few years after diagnosis from the most recent cohort for which such data are available (Figure 3, shaded area).

### Survival trends, the deprivation gap in survival and changes in the deprivation gap

Relative survival up to 10 years was estimated for patients in each of five deprivation categories who were diagnosed in one of the three calendar periods 1986–1990, 1991–1995 and 1996–1999. This set of 15 survival estimates enabled three summary measures to be derived for each cancer and in each sex: the average change in survival over time, the average difference in survival between the most deprived and most affluent groups (the ‘deprivation gap’ in survival), and the average change in the deprivation gap in survival over time. The survival of patients diagnosed during 1986–1990 was used as the baseline for estimates of trend.

We used least-squares linear regression, weighted by the variance of each of the relative survival estimates (Grizzle *et al*, 1969), in Stata software (StataCorp, 2006). The summary measures were estimated by fitting a regression model to the survival estimates with terms for calendar period and deprivation, then adding a further term for interaction between period and deprivation. The significance of the additional term was evaluated with a likelihood ratio test at the 5% level.

The fitted average difference in survival between successive calendar periods, adjusted for socioeconomic differences, is reported in Table 1 of each cancer-specific article. For consistency with earlier work, and because national estimates of survival rates do not generally change very rapidly from year to year, we report the overall national trends in relative survival as the absolute average change every 5 calendar years, adjusted for change in the deprivation gap in survival. Thus an increase from 50 to 55 and 60% in successive 5-year periods would be reported as a 5% increase every 5 years. The same 5-yearly scale is used for trends in the deprivation gap, weighted to take account of the shorter final period (4 years).

**Figure 4:**
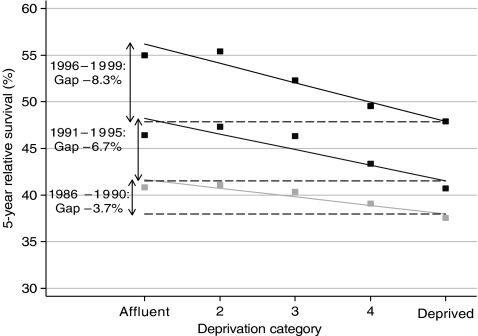
Schema to show how trends in the ‘deprivation gap’ in survival are evaluated: 5-year relative survival, rectal cancer, women diagnosed 1986–1999, England and Wales.

The absolute difference between the relative survival estimates fitted by the regression model for the most affluent and most deprived groups is described as the ‘deprivation gap’ in survival (Figure 4). It is reported as negative if survival was lower for the ‘poorest’ patients than for the ‘richest’ patients. The linear models generally fitted the data well, but the deprivation gap is not simply the difference between survival in the richest and poorest groups: it is a fitted estimate of the difference, incorporating the data for all deprivation groups.

The deprivation gap in each of the three periods is shown in Table 2 in each cancer article, together with the fitted change in the deprivation gap over time. Both estimates are adjusted for time trends in survival within each deprivation group.

Changes in the deprivation gap in survival between successive calendar periods, adjusted for secular trends in survival, were estimated from the interaction between calendar period and deprivation. Thus the deprivation gap for rectal cancer widened from −3.7% for women diagnosed during 1986–1990 to −8.3% for women diagnosed during 1996–1999 (Figure 4), a weighted average widening of −2.5% every 5 years.

These analyses were carried out separately for relative survival at 1, 5 and 10 years after diagnosis, for 16 cancers in men and for 17 cancers in women.

## Discussion

The trends and inequalities in cancer survival in England and Wales presented here for 1986–2001 extend the results for patients diagnosed during the 20-year period 1971–1990 (Coleman *et al*, 1999). For most cancers, the additional analyses show that survival up to 10 years after diagnosis increased for many of the 20 most common cancers in adults, and suggest that the increases in survival are likely to continue in the near future. Despite the overall improvements, however, socioeconomic inequalities in survival for many cancers among adults diagnosed up to 1990 widened further over the period 1986–2001 (Coleman *et al*, 2004).

The analyses reported here are more extensive than those published in 2004. We present estimates and trends in survival up to 10 years after diagnosis. We present the deprivation gap in survival up to 10 years, and trends over time in this deprivation gap at 1, 5 and 10 years after diagnosis. We used the recently developed hybrid approach (Brenner and Rachet, 2004) to improve short-term predictions of survival, because it is unbiased when, as with the data available to us, the incidence data are not as recent as the mortality data. It provides more precise estimates, with narrower confidence intervals, because it includes additional subjects who contribute to the conditional probabilities of relative survival in the period immediately after diagnosis. In that setting, the period approach is unstable. Thus the hybrid approach made it possible, for the first time, to obtain robust short-term predictions of socioeconomic differences in cancer survival up to 10 years after diagnosis for patients diagnosed during 2000–2001.

### Clinical insights into cancer survival patterns

The patients seen by a given clinician are unlikely to be representative of the entire population of cancer patients, but the clinician has a great deal of information about the clinical and personal picture when considering the management and likely outcome for his or her patients.

Epidemiologists handle much more limited data on each patient, but the data are available for virtually all cancer patients, without selection, even patients who are not referred for specialist treatment, and patients who are too sick when diagnosed to be investigated with a view to treatment of curative intent.

These two perspectives of progress in cancer survival are often in sharp but instructive contrast.

For example, the clinical commentary on trends in lung cancer survival (Zee and Eisen, 2008) suggests that comorbidity can influence the choice of surgery or adjuvant chemotherapy or radiotherapy, so that if (or because) comorbidity is more marked in the deprived socioeconomic groups, comorbidity could indirectly influence the deprivation gap in survival. This clinical observation merits further examination, not just for lung cancer but also for cancers of other organs. One approach would be to use data from the Hospital Episodes Statistics system.

Stage at diagnosis is also a contributory factor to these differences in survival: tumour biology and factors related to the healthcare system, such as access to treatment and medical expertise, are also likely to contribute (Kogevinas, 1990; Kogevinas *et al*, 1991; Kogevinas and Porta, 1997; Woods *et al*, 2006).

For most cancers, socioeconomic inequalities in relative survival are no wider at 5 or 10 years after diagnosis than at 1 year, suggesting that most of the difference in excess mortality between rich and poor arises soon after diagnosis. This pattern roughly mimics the pattern seen for elderly patients in England and Wales (Coleman *et al*, 1999; Rachet *et al*, 2005). Elderly patients often have much higher excess mortality than younger patients within the first year or so after diagnosis, but the increased mortality in the first year tends to be greatly attenuated, or to vanish altogether, in the longer term.

Breast cancer is a striking exception to this pattern: deprived women have lower survival than affluent women soon after the diagnosis, but the socioeconomic inequalities in survival actually widen with time since diagnosis, and the deprivation gap in survival at 5 and 10 years is twice as wide as at 1 year after diagnosis. Stage at diagnosis is unlikely to explain this pattern. The most plausible explanation is socioeconomic differences in late recurrence, which in turn could have several causes. Lower take-up of radiotherapy has been shown to lead to higher recurrence (Early Breast Cancer Trialists' Collaborative Group, 2000) and lower survival (Early Breast Cancer Trialists' Collaborative Group, 2005b). Differences in access to chemotherapy or hormonal therapy may also have contributed. Tamoxifen improves long-term survival (Early Breast Cancer Trialists' Collaborative Group, 2005a), and any socioeconomic differences in access to it in the late 1990s could have had an impact on the survival gradient.

A different insight arises from ovarian cancer, for which survival improved steadily over the period 1986–2001, continuing the trend seen in earlier decades, whereas socioeconomic inequalities in survival steadily declined over the same period. Thus 5-year relative survival in England and Wales rose from 21 and 22% in the early and late 1970s, respectively, to 27 and 28% in the early and late 1980s (Coleman *et al*, 1999), up to 31 and 38% for women diagnosed in the early and late 1990s (Cooper *et al*, 2008). For 1-year survival, the deprivation gap remained at about −5%, but for 5-year survival, the deprivation gap shrank in the early 1990s and had disappeared by the late 1990s (+1%). These trends evoked the comment (Kitchener, 2008): ‘The deprivation gap at 1 year can probably be explained by a greater proportion of women in deprived communities having advanced disease, and certainly in the past, poorer access to optimal treatment. This would have resulted in a higher proportion of treatment failures. Better access to specialist treatment has seen this gap close’. He adds that the absence of a deprivation gap in 5-year survival ‘probably also reflects improved access to specialist treatment, and differences in comorbidity between richer and poorer seem not to impact on survival.’

### Interpretation

Most of the clinical commentators have interpreted the improvements in survival over time as being directly attributable to improvements in diagnosis and treatment (particularly surgery). By contrast, they mostly attribute the socioeconomic inequalities in survival to higher levels of comorbidity among the more deprived patients. With some exceptions, socioeconomic inequalities in prompt diagnosis and optimal treatment have not been considered as a potential explanation for socioeconomic differences in survival.

This view is straightforward and inherently plausible, and it derives directly from clinical experience, but it is challenged by some of the results of this study.

Because relative survival estimates are compensated for differences in background mortality among socioeconomic groups, differential co-morbidity can only contribute to socioeconomic differences in relative survival if there is an interaction between the presence of comorbidity and the treatment actually received for the cancer. Survival has been improving for both rich and poor patients, but for many adult cancers, it has been improving more rapidly for the rich than the poor, or at a similar speed for all groups, that is, without closing the deprivation gap in survival.

If differential comorbidity between poor and rich cancer patients were to explain those persistent or widening socioeconomic inequalities in survival, it would imply that the impact of comorbidity on the choice (or the outcome) of treatment had actually been increasing more among the poor than the rich. This seems implausible.

Thus, for cancers of the colon and rectum, socioeconomic differentials in 1-year and 5-year survival widened to 6–9% during the 1990s, at the same time as overall survival for all socioeconomic groups combined improved by 10% or more over the same period (Mitry *et al*, 2008). This is in marked contrast to the pattern for ovarian cancer, for which the steady improvement in survival was accompanied by a consistent narrowing of the deprivation gap in survival. In the face of these patterns, it seems difficult to sustain the view that greater comorbidity in the poor than the rich can somehow underpin persistent socioeconomic inequalities in survival.

Furthermore, the levels of survival among the most affluent patients can in fact be attained by the most deprived. For many cancers, as overall survival has improved, the poorest patients have indeed attained the survival seen for the rich, but often only after a time lag of some 5 years or so (see Figure 4). If the socioeconomic differences in survival for patients diagnosed in a given period were solely attributable to the impact of comorbidity on the choice (or the outcome) of treatment, then the fact that survival in the poor ‘catches up’ with survival in the rich, just a few years later, would imply that the greater comorbidity in poorer cancer patients had somehow vanished over the same period of time. This also seems unlikely, and it is in direct contrast with the earlier argument.

Socioeconomic inequalities in diagnosis or treatment should be seen as a potential alternative cause of differences in cancer survival between social groups.

Racial differences in cancer survival in the United States offer an instructive parallel. Blacks and the socioeconomically disadvantaged are diagnosed at a later stage and have lower survival (Mayberry *et al*, 1995; Bradley *et al*, 2002; Freeman and Reuben, 2002; VanEenwyk *et al*, 2002), and racial disparities in treatment, not explained by clinical factors, are associated with worse outcomes (Shavers and Brown, 2002; Smedley *et al*, 2003). In the equal-access healthcare system run by the Veterans Affairs administration, however, there is very little racial difference in survival among patients managed for colorectal cancer (Rabeneck *et al*, 2003).

In Canada, with universal health insurance, the risk of death within 30 days of hospital admission for stroke was inversely associated with median neighbourhood income, and the excess risk was still observable a year after admission. Patients in the poorest fifth of neighbourhoods had less access to specialist neurologists, and those who had carotid endarterectomy waited much longer than patients in the richest neighbourhoods (Kapral *et al*, 2002). This study is of nonmalignant disease, but it offers a striking example of socioeconomic differences in treatment, care and fatal outcome, in a universal-access healthcare system not unlike that of the United Kingdom.

In the clinical setting, the natural interpretation of differences in cancer survival turns on the adequacy of cancer management by a clinical team or hospital, or in phase III randomised trials, on the efficacy of a new treatment. Population-based cancer survival, by contrast, is a much broader index of the overall performance of healthcare systems (Micheli *et al*, 2003b), but it is often misinterpreted simply as a direct reflection of the competence of the physicians who treat cancer patients (Berrino and Capocaccia, 2008; Haward, 2008). The receipt of inadequate or suboptimal treatment does not necessarily imply poor medical care. It may be the result of differential availability of medical expertise, of policies set by the healthcare providers, of the availability of healthcare resources, or of differential compliance with treatment by the patients themselves. We know, for example, that radiotherapy waiting times have increased (Ash *et al*, 2004), but not whether the impact is greater for patients living in deprived areas.

If we can accept that improvements in national levels of survival are attributable to improved treatment – even in the absence of hard evidence that the better treatments have in fact been made available to all patients – then it seems coherent to accept that differences in survival between subgroups of the population may also be attributable, at least in part, to differences in access to those improved treatments. Inequalities in access to treatment were a key thrust behind the report of the Expert Advisory Group on Cancer (Calman–Hine report) to the Chief Medical Officers of England and Wales in 1995, in the middle of the period covered by these analyses. The report recommended that: ‘all patients should have access to a uniformly high standard of care in the community or hospital, wherever they may live, to ensure the maximum possible cure rates and best quality of life.’ (Expert Advisory Group on Cancer, 1995).

## Summary

Cancer survival in England and Wales is generally improving, but this supplement provides detailed evidence of persistent or widening socioeconomic inequalities in survival for many of the 20 most common types of cancer during the last decade of the 20th century, even after adjustment for socioeconomic differences in background mortality.

These trends occurred during a period when equal access to optimal diagnosis, treatment and care could scarcely have been more prominent on the policy agenda (Expert Advisory Group on Cancer, 1995; Department of Health, 2000, 2003; Cooper, 2001).

Randomised clinical trials provide the crucial evidence that new approaches to the diagnosis or treatment of cancer are capable of improving the outcome for cancer patients. That evidence is often the bedrock for clinical guidelines as to how patients should be investigated and treated. From the broader perspective of public health and cancer control, it is equally important to monitor trends in population-based survival, as an indicator of the extent to which the potential for improved outcomes is actually translated into better survival for all cancer patients.

We welcome the contribution of clinical specialists to explaining both the trends and the persistent socioeconomic inequalities in cancer survival. If this ‘confrontation’ of population-based cancer survival data with expert clinical commentary were to become routine, it might help advance our understanding of the causes of socioeconomic inequalities in survival, and of what might be done to reduce or avoid them. As one clinician recently put it: ‘We know so much about socioeconomic inequalities in survival, yet we do so little’ (Munro, 2005).

The cancer survival patterns reported here strongly suggest that the socioeconomic inequalities in survival can indeed be reduced.

## Figures and Tables

**Table 1:** Ineligible and excluded records, and number (% eligible) of patients included in the analyses: adults (15–99 years) diagnosed with one of twenty common malignancies, England and Wales, 1986–1999

	**No.**	**%**
Total registered	3 018 808	
Ineligible		
Incomplete data^a^	6748	0.2
Patient not resident in England or Wales	14 975	0.5
Tumour *in situ* (behaviour code 2)	322 902	10.7
Tumour benign (behaviour code 0) or uncertain if benign or malignant (1)	35 427	1.2
Metastatic tumour (behaviour code 6 or 9)	9373	0.3
Otherwise ineligible^b^	3909	0.1
	393 334	13.0
Total eligible	2 625 474	100.0
Exclusions from analysis		
Aged 100 years or more at diagnosis	1009	<0.1
Vital status unknown at study closure date	42 019	1.6
Sex not known	2	<0.1
Sex-site incompatibility	1253	<0.1
Invalid dates or invalid sequence of dates	3080	0.1
Zero survival or death certificate only (DCO)	272 607	10.4
Duplicate registration^c^	0	0.0
Synchronous tumours^d^	12 713	0.5
Multiple primary at the same site^e^	5262	0.2
Multiple primary at a different site^e^	79 664	3.0
	417 609	15.9
Patients accepted for analyses	2 207 865	84.1

a Main data item(s) invalid or incompatible with one another: sex, date of birth, date of diagnosis and (if present) date of death, postcode, site, morphology and behaviour.

b Other criteria of anatomic location, morphology or behaviour, specific to a particular malignancy.

c Same site code, sex, cancer registry and cancer registry number as an earlier registration.

d Same site code, sex, date of birth and date of diagnosis as another registration(s): mostly synchronous or (in paired organs) bilateral tumours in same anatomic site in one individual, not linked earlier: also some duplicate registrations.

e Same site code and person number as an earlier registration(s): mostly confirmed multiple primary tumours at the same or a different anatomic site, some unresolved duplicate registrations.
